# *Mycobacterium bolletii* Respiratory Infections

**DOI:** 10.3201/eid1502.080837

**Published:** 2009-02

**Authors:** Toïdi Adékambi, Michel Drancourt

**Affiliations:** Université de la Méditerranée, Marseille, France; 1 Current affiliation: Centers for Disease Control and Prevention, Atlanta, Georgia, USA.

**Keywords:** Mycobacterium bolletti, M. chelonae-abscessus group, respiratory infections, dispatch

## Abstract

Contrary to other species in the *Mycobacterium chelonae-abscessus* complex, we reidentified *M. bolletii* strains isolated from 4 respiratory patients and found these strains to be uniformly resistant to clarithromycin. No mutations previously associated with macrolide resistance in bacteria were detected in either the 23S rDNA or the genes encoding riboproteins L4 and L22.

*Mycobacterium chelonae-abscessus* complex (MCAC) members are opportunistic pathogens in patients with underlying pulmonary disorders (*1*–*3*). We recently described *M. bolletii* as a new MCAC member (*4*); the pathogen was later isolated from 9% of cystic fibrosis patients (*5*). The initial description of *M. bolletii* suggested that it was highly resistant to antimicrobial drugs, including clarithromycin (*4*). To gain a better appreciation of this resistance, we reidentified MCAC isolates collected in our microbiology laboratory, Timone Hospital, Marseilles, during the past 10 years and performed in vitro susceptibility testing and sequencing of the 23S rDNA, L4, and L22 genes.

## Case Reports

Patient 1, a 47-year-old woman with an unremarkable medical history, sought treatment after a 3-week history of hemoptysis; radiographs showed bilateral micronodular infiltrates in the upper lung lobes. Blood tests showed a polymorphonuclear cell count of 9 × 10^3^/mL and biological inflammatory syndrome. Bleeding in the left lobar bronchus was observed during bronchoscopy. Microbiologic results of a bronchial lavage specimen were negative, and microscopic examination found no acid-fast bacilli. An isolate from a subsequent sputum sample was identified later as *M. bolletii*, however. The patient was discharged. No further information is available for this patient.

Patient 2, a 76-year-old man who had received treatment for pulmonary tuberculosis (TB) in 1976, sought treatment in November 1995 for hemoptysis and signs and symptoms of broncho-pulmonary infection. A chest radiograph showed a large cavity in the right upper lobe and infiltrate in the left upper lobe. Two stomach aspirates yielded an isolate identified as *M. abscessus* despite negative results of a direct microscopic examination; refined identification performed 8 years later in 2003 found both isolates to be *M. bolletii*. The patient received rifampin, clarithromycin, isoniazid, and ciprofloxacin for 16 months. After an initial improvement, the patient continued to have an episodic cough and hemoptysis, and *M. bolletii* was grown from 2 sputum specimens; direct microscopic examination yielded acid-fast bacilli. In November 1998, the patient still had symptoms. In 2003, he was admitted to an intensive care unit for acute respiratory distress syndrome. A broncho-alveolar lavage specimen yielded a mixed culture of multidrug-resistant *M. bolletii* and *Klebsiella pneumoniae* despite negative results of direct examination. The patient eventually died of *K. pneumoniae* septicemia within days of his admission to the ICU.

Patient 3, a 77-year-old man who smoked 3 packs of cigarettes per month and who had a history of pulmonary TB, was admitted to Timone Hospital, Marseilles, in October 2000 with a diagnosis of bronchitis. Corticoid therapy was prescribed. In March 2001, the patient was admitted for respiratory insufficiency. A chest radiograph showed diffuse bullous emphysema in both lungs. Microscopic examination of 1 sputum specimen and 1 bronchial aspirate yielded the presence of acid-fast bacilli that were later identified as *M. bolletii*. The patient left the hospital without treatment, and no further information on his condition is available.

Patient 4, a 90-year-old woman, sought treatment with a temperature of 38°C, hemoptysis, and bilateral micronodular infiltrates of the upper lung lobes. She had a history of childhood pulmonary TB. Sputum specimens yielded mycobacteria that were identified later as *M.* *bolletii* despite negative results of direct examination. The patient remained febrile after 1 month of treatment with intravenous imipenem and amikacin, her pulmonary condition worsened, and a sputum smear showed numerous acid-fast bacilli. The treatment regimen was changed to a combination of ciprofloxacin, clarithromycin, and ethambutol, but the patient died 2 weeks after treatment began.

## The Study

Thirty-one isolates, previously identified as *M. abscessus* (n = 20) and *M. chelonae* (n = 11) by 16S rDNA sequencing from January 1996 through June 2007, were reidentified by partial *rpoB* gene sequencing as described (*6*). The MICs of 22 antimicrobial drugs were determined by E-test (AB Biodisk, Solna, Sweden) after the culture was incubated for 3 days at 30°C. Data were interpreted by using the broth microdilution criteria (*7*). A 1,500-bp fragment of the 23S rDNA (*8*), a 635-bp fragment of the L4 ribosomal protein (forward primer L4Absc7F: 5′-ATCGCAGTCAAGGCTCCGG-3′, reverse primer L4Absc642R: 5′-TCAGGCCGACACCTCCTC-3′), and a 471-bp fragment of the L22 ribosomal protein (forward primer L22Absc1F: 5′-ATGACCACTACTACCGAAT-3′, reverse primer L22Absc471R: 5′-CTAGCTGGTGCCTCCCTT-3′) were PCR amplified and sequenced from clarithromycin-resistant isolates. The study was approved by local Ethics Committee, Marseilles Medical School.

Twelve of 31 isolates were identified by *rpo*B sequencing as *M. abscessus*, 11 as *M. chelonae*, 4 as *M. massiliense,* and 4 as *M. bolletii.* *M. abscessus* and *M. bolletii* isolates showed no intraspecific *rpoB* sequence variation. In contrast, 0.7% sequence divergence was observed in *M. chelonae* (6 sequevars) and *M. massiliense* (3 sequevars) isolates (Figure). The 4 *M. bolletii* isolates were unique among these 31 MCAC isolates in that they were multidrug resistant (Table). The isolates exhibited clarithromycin MICs >256 µg/mL, whereas the other MCAC isolates had clarithromycin MICs <2 µg/mL. The E-test is not a validated method for MIC determination in rapidly growing mycobacteria, yet the results we obtained were similar to those previously reported for the reference broth microdilution method (*9*). In the *M. bolletii* isolates, we found no substitutions, deletions, or insertions in domain V (A2058, A2059, C2611 position, *Escherichia coli* numbering) of the 23S rDNA or in the L4 and L22 ribosomal protein genes.

**Figure:**
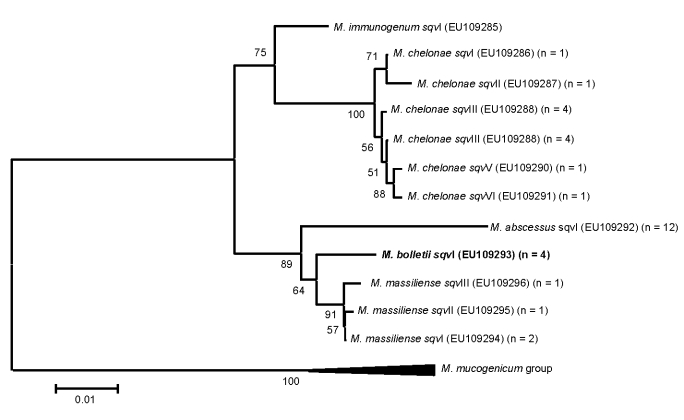
Phylogenetic tree of different sequevars of *Mycobacterium abscessus-chelonnae* group isolates determined by *rpo*B-723 bp sequencing. Isolate identified as *M. bolletii* is in **boldface**. Scale bar represents 1% sequence divergence.

**Table:** Antimicrobial drug susceptibility test results (MICs) for *Mycobacterium bolletii* isolates*

Antimicrobial agent	Patient 1	Patient 2	Patient 3	Patient 4
Penicillin	>32	>32	>32	>32
Amoxicillin	>256	>256	>256	>256
Amoxicillin clavulanate (disc 20 µg + 10 µg)	>4	>4	>4	>4
Cefoxitin	>256	>256	>256	>256
Ceftriaxone	>256	>256	>256	>256
Cefotaxime	>256	>256	>256	>256
Imipenen	>32	>32	>32	>32
Doxycycline	>32	>32	>32	>32
Minocycline	>256	>256	>256	>256
Clarithromycin	>256	>256	>256	>256
Erythromycin	>256	>256	>256	>256
Azithromycin	>256	>256	>256	>256
Amikacin	32	48	32	48
Tobramycin	>32	>32	>32	>32
Ciprofloxacin	>32	>32	>32	>32
Ofloxacin	>32	>32	>32	>32
Sparfloxacin	>32	>32	>32	>32
Rifampicin	>32	>32	>32	>32
Metronidazole	>256	>256	>256	>256
Teicoplanine	>256	>256	>256	>256
Vancomycin	>256	>256	>256	>256
Trimethoprim-sulfamethoxazole	>32	>32	>32	>32

*Values given in μg/mL.

## Conclusions

*M. bolletii* is an emerging pathogen responsible for respiratory tract infections in patients with underlying compromised respiratory function. In our study, *M. bolletii* was responsible for pulmonary infection in 3 of 4 patients (patients 2–4) (*10*). *M. bolletii* was repeatedly isolated from different samples from those 3 patients over a period of several weeks. A coexisting broncho-pulmonary disease was found in 3 of the 4 patients, and clinical features and radiograph patterns that suggested nontuberculous mycobacteria infection were observed in all 4 patients (*10*). Infected patients were >75 years of age. Three of 4 patients had hemoptysis and lung infiltrates, *M. bolletii* was the sole organism isolated from respiratory tract specimens. In this study, 16S rDNA sequencing misidentified 25% of 31 MCAC isolates that were eventually identified as *M. massiliense* and *M. bolletii*. This statistic agrees with observations made during the recent description of *M. bolletii* infection after mesotherapy (*11*).

Clarithromycin was administrated to 2 of the 4 patients, both of whom died within several weeks after treatment began. The 4 *M. bolletii* isolates were highly resistant to clarithromycin, yet they did not harbor the 23S rDNA mutations that have been previously found in clarithromycin-resistant *M. abscessus* strains (*12*). Additionally, no mutations in riboproteins L4 and L22, which are associated with macrolide resistance in *Streptococcus pneumoniae* (*13*), were detected. RNA methylase genes *erm*[38], *erm*[39], and *erm*[40], which confer inducible macrolide resistance in *M. fortuitum*, *M. smegmatis*, *M. mageritense*, and *M. wolinskyi,* are absent in the MCAC (*14*). Further investigations are therefore needed to clarify the mechanism of clarithromycin resistance in *M. bolletii*.

Clarithromycin has been recommended as the first-line antimicrobial drug for treating rapidly growing mycobacteria infections in patients with compromised respiratory function (*1*,*10*). Patient deaths (*3*) have been linked to clarithromycin resistance, with a risk of secondary clarithromycin resistance during monotherapy estimated to be <10% (*12*). Recent studies showed that 21%–36% of MCAC isolates were resistant to clarithromycin (*15*). The recommendation for treating *M. abscessus* infection with clarithromycin was made before discovering *M. bolletii*, a multidrug-resistant species that mimics *M. abscessus*. Our report illustrates that accurate species identification and in vitro clarithromycin susceptibility testing should be recommended for MCAC isolates of clinical interest. GenBank accession nos. were as follows: for 23S rDNA sequences, *M. bolletii* CIP 108541^T^ (EU109306), *M. massiliense* CIP 108297^T^ (EU109307), *M. abscessus* CIP 104536^T^ (EU109308), and *M. chelonae* CIP 104535^T^ (EU109309); for L4 sequences, *M. bolletii* CIP 108541^T^ *M. massiliense* (EU779956), CIP 108297^T^ (EU779957), *M. abscessus* CIP 104536^T^ (EU779957), and *M. chelonae* CIP 104535^T^ (EU779958); and for L22 sequences *M. bolletii* CIP 108541^T^ (EU779952), *M. massiliense* CIP 108297^T^ (EU779953), *M. abscessus* CIP 104536^T^ (EU779954), and *M. chelonae* CIP 104535^T^ (could not be amplified with the designed primers).
